# A dog presenting with syncope due to two different etiologies

**DOI:** 10.5455/javar.2022.i612

**Published:** 2022-09-30

**Authors:** Mizuki Ogawa, Hirosumi Miyakawa, Huai-Hsun Hsu, Yuichi Miyagawa, Naoyuki Takemura

**Affiliations:** Laboratory of Veterinary Internal Medicine II, School of Veterinary Medicine, Nippon Veterinary and Life Science University, Musashino-Shi, Japan

**Keywords:** Cilostazol, pimobendan, pulmonary hypertension, sick sinus syndrome, sinus arrest

## Abstract

**Objective:**

The treatment of syncope depends largely on its possible etiology. Therefore, identifying the cause of syncope is very important in treatment planning. Herein, we report an etiology of syncope caused by pulmonary hypertension (PH) associated with canine filariasis, followed by syncope due to bradyarrhythmia 1 year later.

**Materials and Methods:**

An 8-year-old male English Cocker Spaniel was referred to our hospital for a second opinion regarding syncope that the dog had started experiencing approximately 2 months prior. Based on the examination findings, we diagnosed that the fainting was due to heartworm disease and associated PH. After increasing the dose of pimobendan (0.50 mg/kg, q12h), the syncope subsided. However, syncope recurred on the 215th day of the first episode.

**Results:**

The findings that differed from those during the initial examination were that cardiac arrest was observed for approximately 5 sec during auscultation, along with sinus arrest. Therefore, to further investigate for syncope, a Holter electrocardiograph was obtained for 3 days. Consequently, sinus arrest was identified as the etiology of the recurrent syncope, and the patient was diagnosed with sick sinus syndrome, Rubenstein classification type II. Following cilostazol (10 mg/kg, q12h) administration, the syncope subsided.

**Conclusion:**

This case reports syncope in a dog, which typically occurs due to different etiologies. When a dog has PH, it may be important to think about the possibility of arrhythmias caused by a bigger right heart.

## Introduction

Reduced cardiac output is a possible etiology of syncope in dogs. Identifying the cause of syncope is very important in the formulation of a treatment strategy, as diseases with different etiologies have different treatment regimens [1]. The heartworm infection, caused by canine filariasis (*Dirofilaria immitis*) parasites in the pulmonary arteries, may result in syncope due to decreased blood perfusion to the brain caused by decreased cardiac output [2]. Additionally, the complication of pulmonary hypertension (PH) in this disease may exacerbate syncope. Recently, arrhythmias have been reported in human patients with PH [3]. Dogs often faint due to either PH or arrhythmia [1,4], but those that experience both PH and arrhythmia-induced syncope are rare. To our knowledge, there are no case reports of syncope due to arrhythmia secondary to PH in dogs. We report a case of syncope caused by PH associated with canine filariasis, followed by syncope due to bradyarrhythmia 1 year later.

## Case Presentation

An 8-year-old, 15.8 kg male English Cocker Spaniel was referred to our hospital for a second opinion regarding syncope that the dog had started experiencing approximately 2 months prior. Appetite and activity were normal and no clinical signs other than syncope were observed. Syncope was observed only during excitement. As it was a shelter dog, its medical history was unknown.

## Clinical Examination (First Round)

During the initial examination, the heart rate was 72 beats/min and Levine 3/6 systolic regurgitation murmurs were heard from the bilateral chest wall. The dog had a rectal temperature of 38.2°C and a body condition score of 4/9.

Hematology and serum biochemistry revealed no abnormal findings; however, the canine heartworm antigen test was positive.

**Figure 1.**
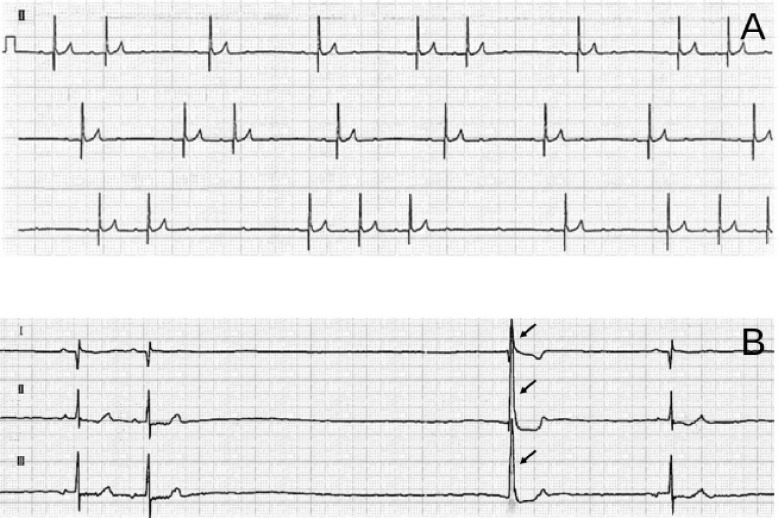
ECG of the first round (A) and second round (B). (A) During the initial examination, the heart rate is 47–51 beats/min with bradycardia. Furthermore, two-lead ECG tracing shows first- and second-degree atrioventricular blocks (Mobitz type II). In the approximately 5-min ECG recording, other than sinus arrhythmia, no other arrhythmias are identified. Paper speed, 25 mm/sec; 1 cm, 1 mV. (B) As in the initial examination, three-lead ECG tracing during recurrent syncope (at the second visit) shows sinus arrhythmia and first- and second-degree atrioventricular blocks (Mobitz type II). The heart rate is normal at 80–120 beats/min. However, three-lead ECG tracing shows sinus arrest lasting more than thrice the normal P–P interval. The period of sinus arrest includes a junctional escape beat (arrows). The duration of sinus arrest is 2.5 sec, during which no syncope is observed. Paper speed, 50 mm/sec; 1 cm, 1 mV; ECG, electrocardiograph.

The electrocardiograph (ECG) revealed bradycardia with a heart rate of 47–51 beats/min, indicating sinus arrhythmia. Furthermore, a two-lead ECG trace revealed first- and second-degree atrioventricular blocks (Mobitz type II; Fig. 1A). In an approximately 5-min ECG recording, we observed no arrhythmias besides sinus arrhythmia.

**Figure 2.**
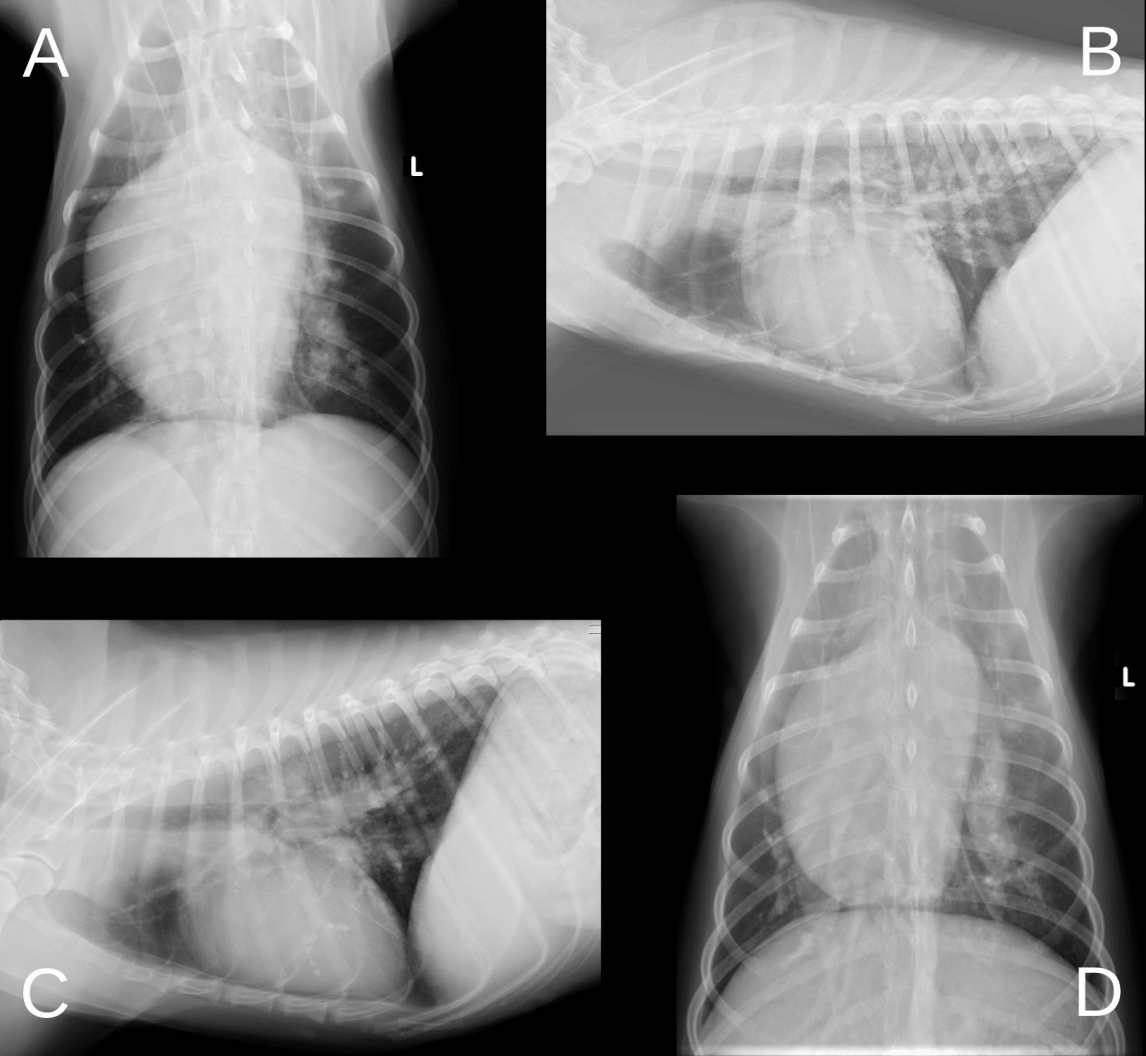
Radiographic images of the first round (A–B) and second round (C–D). (A–B) Reverse “D” shape and truncated peripheral intralobar and interlobar branches of the pulmonary arteries are observed. Moreover, VHS and VLAS are 11.7 v and 2.2 v, respectively. (C–D) VHS and VLAS are 11.1 v and 2.3 v, respectively. There are no significant changes in the pulmonary opacity, compared with the initial examination. VHS, vertebral heart size; VLAS, vertebral left atrial size.

On thoracic radiography, right heart enlargement (reverse “D” shape) and truncated peripheral intralobar and interlobar branches of the pulmonary arteries were identified (Fig. 2A). Moreover, the vertebral heart size (VHS) and vertebral left atrial size (VLAS) measured in the lateral view exceeded the reference values of 11.7 and 2.2 v, respectively (Fig. 2B).

**Figure 3.**
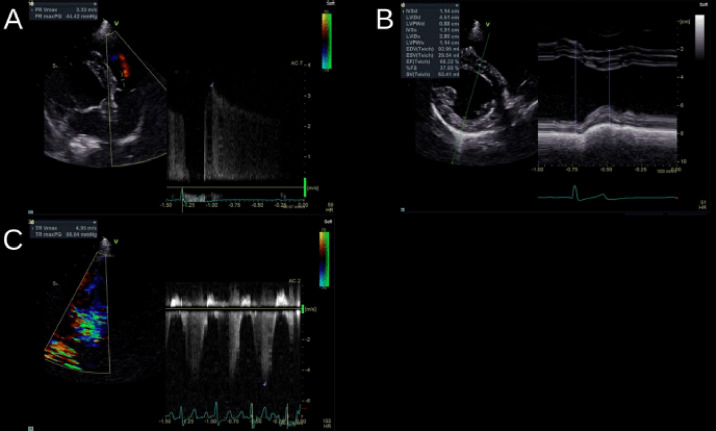
Radiograph images of the first round (A–B) and second round (C). (A–B) The pulmonary valve regurgitation (3.33 m/sec) is observed, and the left intraventricular space is compressed due to ventricular septal flattening. (C) The tricuspid valve regurgitation (4.95 m/sec) is observed, unlike at the initial examination.

The echocardiography suggested no left cardiac enlargement; however, pulmonary valve regurgitation (3.33 m/sec) was observed, and the left intraventricular space was compressed due to ventricular septal flattening (Fig. 3A and B).

## Treatment (First Round)

Based on the aforementioned findings, we diagnosed that the syncope was due to heartworm disease and associated PH [1,2].

Of the arrhythmias present on initial examination, atrioventricular block or bradycardia was the most likely cause of syncope. However, the ventricular rates were more than 45 beats/min, and although bradycardia was present, the patient did not experience syncope during the examination. The patient was expected to experience more severe bradycardia during syncope. However, since the initially observed syncope had occurred during excitement, it was not attributed to the arrhythmias but to heartworm disease and associated PH.

Pimobendan (0.25 mg/kg, PO, bid) was already administered by the referring veterinarian; however, when the dose was increased to 0.50 mg/kg, PO, bid, the syncope disappeared. Thereafter, the patient received regular checkups at a referring veterinarian; however, syncope recurred on the 215th day, after the first episode. It was observed at rest rather than during excitement. The pimobendan dose was further increased by the referring veterinarian (0.64 mg/kg, PO, bid), but the syncope did not improve; therefore, the patient was brought to our hospital on the 336th day.

## Clinical Examination (Second Round)

The heart rate was 80–120 beats/min and Levine 3/6 systolic regurgitation murmurs were heard from the bilateral chest wall. Moreover, cardiac arrest was observed for approximately 5 sec during auscultation.

ECG revealed sinus arrest in addition to first- and second-degree atrioventricular blocks (Mobitz Type II) (Fig. 1B).

On thoracic radiography, VHS and VLAS were 11.1 and 2.3 v, respectively, similar during the initial examination (Fig. 2C). There were no significant changes in the pulmonary opacity compared to during the initial examination (Fig. 2D).

Similar to the findings during the initial examination, ECG showed pulmonary valve regurgitation (3.67 m/sec) and ventricular septal flattening. However, unlike during the initial examination, tricuspid valve regurgitation (4.95 m/sec) was observed (Fig. 3C).

## Treatment (Second Round)

Two possible causes of syncope observed on the 215th day were PH progression and bradyarrhythmia. However, the findings that differed from those during the initial examination were that cardiac arrest was observed for approximately 5 sec during auscultation and that the syncope occurred at rest rather than during excitement. Furthermore, sinus arrest had not been observed during the initial examination. Therefore, Holter ECG was taken for 3 days to further investigate the syncope.

**Figure 4.**
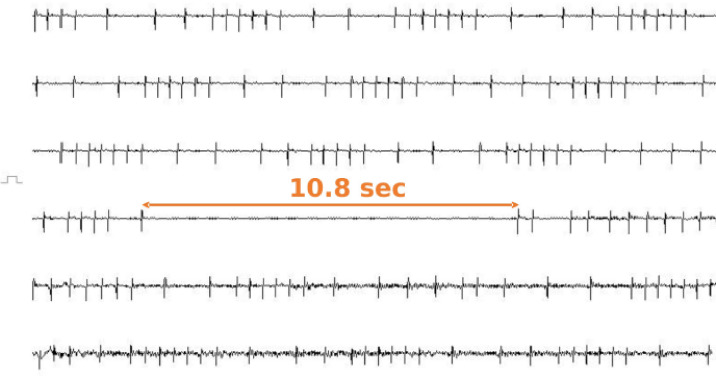
Based on the 336th day’s findings, Holter ECG is taken for 3 days. The figure shows the ECG during syncope. The duration of the sinus arrest coincided with the timing of the syncope, and a 10.8-sec sinus arrest is observed. The heart rate before and after sinus arrest is 78–96 beats/min, neither tachycardic nor bradycardic. Based on the aforementioned findings, we conclude that the cause of syncope in this case is sinus arrest and diagnose the patient with SSS, Rubenstein classification type II. Furthermore, Holter ECG, other than during syncope, shows atrial premature complex and sinus arrhythmias, and the resting heart rate is 50 beats/min with a tendency towards bradycardia. In addition to this figure being recorded using a Holter ECG, it is a compressed figure of 120 sec. Each line is 20 sec. ECG, electrocardiograph.

Syncope was observed five times during the 3-day Holter ECG recording. The ECGs during each syncope revealed sinus arrest (Fig. 4), which was consistent with the description of syncope in the behavioral records. The duration of sinus arrest without syncope was 3.4 sec (range = 2.8–5.2 sec), whereas that of sinus arrest with syncope was 8.5 sec (range = 6.9–10.8 sec). Furthermore, the heart rate before and after sinus arrest during syncope was within the normal range (75–120 beats/min). Therefore, we concluded that sinus arrest caused the recurrent syncope, and the patient was diagnosed with sick sinus syndrome (SSS), Rubenstein classification type II [5]. Therefore, in addition to pimobendan, cilostazol (10 mg/kg, PO, bid) was added for the treatment of SSS. Consequently, the syncope subsided and has not recurred to date.

## Discussion

While various etiologies of syncope in dogs have been reported, there are few reports of syncope due to multiple etiologies in a single patient. In addition, this is the first case report of a dog suffering from PH, followed by secondary bradyarrhythmia and syncope by SSS.

Based on the echocardiography findings, PH was diagnosed according to the most recent consensus statement provided by the American College of Veterinary Internal Medicine [6]. Since heartworm disease was considered to have caused the PH in the current case [7,8], it could be classified as pre-capillary PH Group 4 (thromboembolic PH) [6]. In humans, about half of the patients with thromboembolic PH have syncope [9]. This case also had bradyarrhythmia during the first visit. Dogs with heartworm disease may have arrhythmia due to severe PH [10,11]. The syncope subsided after increasing the pimobendan dose, suggesting that the etiology of the initial syncope was not arrhythmia but a decrease in cardiac output due to PH. The recurrence of syncope at the second visit might have been caused by the deterioration of PH and by bradyarrhythmia. However, unlike during the initial examination, the recurrent syncope occurred at rest, not during excitement, and sinus arrest was observed on the three-lead ECG tracing. Therefore, although PH was confirmed during the second visit, it was not the only etiology of syncope. Moreover, Holter ECG confirmed sinus arrest during syncope, resulting in the diagnosis of SSS.

In most cases, SSS is idiopathic [12]. However, in the current patient, right heart enlargement associated with PH was thought to be related to the development of SSS. In humans with PH, progressive enlargement of the right heart reportedly causes sinus node stretching and associated ischemia or fibrosis, leading to arrhythmias [13]. Tachycardia accounts for most arrhythmias observed in humans with PH; however, bradycardia may occur in some patients [14]. Therefore, we hypothesized that worsening of the right heart enlargement due to the progression of PH and associated ischemia, or fibrosis of the sinus node, may be associated with different causes of syncope during the initial examination and re-examination. Arrhythmias secondary to PH should also be considered in the differentiation of syncope in dogs with PH.

In this case, Holter ECG was performed, which assisted in diagnosing SSS. Although confirming abnormalities in the sinus node recovery time or sinus conduction time by overdrive pacing is necessary to definitively diagnose SSS, in practice, clinical diagnosis is often based on ECG findings when clinical signs manifest [15]. In the current patient, sinus arrest was observed in the hospital during re-examination; however, no syncope was observed then. Syncope due to bradyarrhythmia is not usually identified via ECG examinations performed in the hospital because, in many cases, the animal is in a tense or excited state [16]. The present patient’s in-hospital heart rate was 80–120 beats/min, whereas the resting heart rate at home was 50 beats/min, with a tendency toward bradycardia. In cases with syncope, Holter ECG should be considered if bradyarrhythmia is observed, even if the syncope was not confirmed during ECG examination in the hospital.

Pacemaker implantation is a surgical treatment for SSS [17]. However, because of the PH in the patient, in this case, the risk of administering anesthesia was high; thus, pacemaker implantation was deemed unsuitable. Some dogs with SSS may respond to atropine or oral positive psychotropic drugs, and an atropine stress test may be useful in selecting a treatment [18]. We did not perform atropine loading because of the owner’s refusal. We chose cilostazol (10 mg/kg, q 12 h) as the treatment for SSS [19]. Cilostazol is an oral selective phosphodiesterase type III inhibitor used as an antiplatelet and antithrombotic agent for patients with peripheral arterial occlusive disease [20]. Cilostazol has positive chronotropic effects and has been used to treat human bradyarrhythmia patients, such as atrioventricular block and SSS [21]. The mechanisms of action of cilostazol include vasodilation resulting in a reflex increase in the heart rate, increased blood flow to the sinus node resulting from coronary artery dilatation, and increased sinus node intracellular cyclic adenosine monophosphate [22]. Therefore, cilostazol was considered useful in SSS. However, as the occurrence of bradyarrhythmia in the patient might also have been PH, sildenafil (a pulmonary artery dilator) may be added in the future if cilostazol alone is inadequate to control the syncope [15].

## Conclusion

In this case, right heart enlargement associated with PH led to the development of SSS. In dogs with PH, the possibility of arrhythmias secondary to right heart enlargement may require consideration. In cases of PH with syncope, where treatment is unsuccessful, syncope secondary to arrhythmia may also be considered.

## List of Abbreviations

ECG, electrocardiograph; PH, PO, per os; pulmonary hypertension; SSS, sick sinus syndrome; VHS, vertebral heart size; VLAS, vertebral left atrial size.

## References

[1] Thompson MS (2018). Small animal medical differential diagnosis.

[2] American Heartworm Society (2018). Current canine guidelines for the prevention, diagnosis, and management of heartworm (*Dirofilaria immitis*) infection in dogs.

[3] Reddy SA, Nethercott SL, Khialani BV, Grace AA, Martin CA (2021). Management of arrhythmias in pulmonary hypertension. J Interv Card Electrophysiol.

[4] Johnson LR, Stern JA (2020). Clinical features and outcome in 25 dogs with respiratory-associated pulmonary hypertension treated with sildenafil. J Vet Intern Med.

[5] Willis R, Oliveira P, Mavropoulou A (2018). Guide to canine and feline electrocardiography.

[6] Reinero C, Visser LC, Kellihan HB, Masseau I, Rozanski E, Clercx C (2020). ACVIM consensus statement guidelines for the diagnosis, classification, treatment, and monitoring of pulmonary hypertension in dogs. J Vet Intern Med.

[7] Ames MK, Atkins CE (2020). Treatment of dogs with severe heartworm disease. Vet Parasitol.

[8] Serrano-Parreno B, Carreton E, Caro-Vadillo A, Falcón-Cordón S, Falcón-Cordón Y, Montoya-Alonso JA (2017). Pulmonary hypertension in dogs with heartworm before and after the adulticide protocol recommended by the American Heartworm Society. Vet Parasitol.

[9] Imtiaz S, Cadwelding AI, Alqahtani NH, Idrees MM (2021). Clinical and physiological characteristics of, medically treated, chronic thromboembolic pulmonary hypertension patients in Saudi Arabia: a single center experience. Ann Thorac Med.

[10] Romano AE, Saunders AB, Gordon SG, Wesselowski S (2021). Intracardiac heartworms in dogs: clinical and echocardiographic characteristics in 72 cases (2010-2019). J Vet Intern Med.

[11] Maerz I (2020). Clinical and diagnostic imaging findings in 37 rescued dogs with heartworm disease in Germany.

[12] Burrage H (2012). Sick sinus syndrome in a dog: treatment with dual-chambered pacemaker implantation. Can Vet J.

[13] Bandorski D, Höltgen R, Ghofrani A, Johnson V, Schmitt J (2019). Herzrhythmusstörungen bei patienten mit pulmonaler hypertonie und lungenerkrankungen [Arrhythmias in patients with pulmonary hypertension and chronic lung disease]. Herzschrittmacherther Elektrophysiol.

[14] Hoeper MM, Galié N, Murali S, Olschewski H, Rubenfire M, Robbins IM (2002). Outcome after cardiopulmonary resuscitation in patients with pulmonary arterial hypertension. Am J Respir Crit Care Med.

[15] Ward JL, DeFrancesco TC, Tou SP, Atkins CE, Griffith EH, Keene BW (2016). Outcome and survival in canine sick sinus syndrome and sinus node dysfunction: 93 cases (2002–2014). J Vet Cardiol.

[16] Petrie JP (2005). Practical application of holter monitoring in dogs and cats. Clin Tech Small Anim Pract.

[17] Johnson MS, Martin MWS, Henley W (2007). Results of pacemaker implantation in 104 dogs. J Small Anim Pract.

[18] Thomason JD, Fallaw TL, Calvert CA (2008). ECG of the month. J Am Vet Med Assoc.

[19] Fukushima R, Kawaguchi T, Yamada S, Yoshimura A, Hirao D, Oomori T (2018). Effects of cilostazol on the heart rate in healthy dogs. J Vet Med Sci.

[20] Noma K, Higashi Y (2018). Cilostazol for treatment of cerebral infarction. Expert Opin Pharmacother.

[21] Moriya I, Takahashi T, Nomura Y, Kawaura K, Kusaka K, Yamakawa J (2004). Chronotropic effect of the antithrombotic agent cilostazol in a patient with sick sinus syndrome and syncope. J Int Med Res.

[22] Nimura A, Sato N, Sakuragi H, Koyama S, Maruyama J, Talib AK (2011). Recovery of advanced atrioventricular block by cilostazol. Intern Med.

